# Chronic Myeloid Leukemia and the T315I *BCR::ABL1* Mutation

**DOI:** 10.3390/ijms262311285

**Published:** 2025-11-21

**Authors:** Federico Pierro, Stefania Stella, Manlio Fazio, Sabina Russo, Michele Massimino, Giuseppe Mirabile, Daniela Belletti, Alessandro Allegra, Fabio Stagno

**Affiliations:** 1 Division of Hematology, Department of Human Pathology in Adulthood and Childhood “Gaetano Barresi”, University of Messina, Via Consolare Valeria, 98125 Messina, Italy; federico.pierro@studenti.unime.it (F.P.); manliofazio@hotmail.it (M.F.); sabina.russo@polime.it (S.R.); giuseppe.mirabile@polime.it (G.M.); aallegra@unime.it (A.A.); 2 Department of Clinical and Experimental Medicine, University of Catania, 95123 Catania, Italy; stefania.stella@unict.it; 3 Department of General Surgery and Medical-Surgical Specialties, University of Catania, 95123 Catania, Italy; michedot@yahoo.it; 4 IRCCS Bonino Pulejo Center, 98124 Messina, Italy; dany.11febbraio@libero.it

**Keywords:** chronic myeloid leukemia, *BCR::ABL1*, CML therapy, T315I mutation, CML mutation, prognosis, acquired resistance

## Abstract

Chronic myeloid leukemia (CML) is a myeloproliferative neoplasm characterized by both an abnormal expansion of the granuloblastic clone and the pathognomonic presence of the Philadelphia (Ph) chromosome that generates the BCR::ABL1 oncoprotein. Despite the surfacing of tyrosine kinase Inhibitors (TKIs) in 2001, which changed the evolution of the disease, resistance due to point mutation or compound alteration during treatment with target therapy may occur. One of the mutations that is still an on-going challenge in clinical and scientific field is the T315I mutation, since it gives patients a poor prognosis attributable to acquired resistance to therapy. In the following narrative review, we will discuss the current knowledge on the T315I mutation, explore the most suitable treatment options, examine the role of third-generation tyrosine kinase inhibitors, and outline potential future therapeutic strategies.

## 1. Introduction

Chronic Myeloid Leukemia (CML) is a clonal myeloproliferative disease characterized by an abnormal expansion of the granuloblastic clone and by the pathognomonic presence of the Philadelphia (Ph) chromosome that arises from a reciprocal translocation between chromosomes 9 and 22 [1,2]. This chromosomal translocation produces the formation of a distinct chimeric BCR-ABL fusion gene, which has a pivotal role in causing the leukemic transformation of the hematopoietic stem cells and is also responsible for the further transformations of the CML clone [3,4].

The Ph chromosome is detected in approximately 95% of CML cases and 15–20% of adult acute lymphoblastic leukemia (ALL) [3,5,6]. In the remaining 5% of CML cases, the *BCR::ABL1* fusion gene is considered “cryptic”, residing on a morphologically normal chromosome 22 or, less frequently on chromosome 9 [7]. Different *BCR::ABL1* fusion transcripts can be produced depending on the *BCR* and *ABL1* exons involved. The most frequent *BCR::ABL1* isoforms include e13a2 and e14a2 (both p210) [8], while rare *BCR::ABL1* transcripts such as e1a2, e19a2, e13a3, and e14a3 were also reported [9,10]. Furthermore, most ALL patients express p190 BCR::ABL1 in B lymphoid precursors and previous data have shown that some of these patients may also express p210 BCR-ABL1 or co-express p190 and p210 [11].

The identification of the Ph chromosome in 1960 represented a pivotal milestone, establishing the first direct association between a chromosomal abnormality and a specific type of cancer. Consequently, CML has become a historical model of disease in oncology, from its initial description in the mid-19th century to its transformation into one of the first cancers successfully treated [12]. According to the World Health Organization criteria, CML follows a biphasic clinical course: a chronic phase (CP) followed by a blastic phase (BP) [13]. Many patients are initially diagnosed in CP, a stage of the disease in which symptoms are generally manageable. However, in the absence of effective medical intervention, the disease may evolve through a state of progressive disease (previously named accelerated phase: AP), characterized by increasing hematologic instability and eventually culminating in a BP, which resembles acute leukemia [2]. The transformation from CP-CML into the BC is marked by a loss of cellular differentiation and peculiar gene expression profiles [14]. This advanced stage is characterized by the accumulation of immature blast, which suppress the production of normal hematopoietic elements. Blast crisis is typically highly resistant to therapy and mortality often results from infections or hemorrhagic complications due to the severe depletion of functional granulocytes and platelets.

Moreover, this phase is driven by secondary molecular and cytogenetic alterations, ultimately leading to the clonal evolution and a blockade of normal cellular differentiation [3,15,16,17].

Over the past two decades, the introduction of the first- (1G), second- (2G) and third- (3G) semi-specific *BCR::ABL1* tyrosine kinase inhibitor (TKI) has significantly improved the outcome of patients with CP CML, achieving unprecedented rates of hematologic, cytogenetic and molecular response [18,19,20]. Despite these remarkable outcomes, a subset of patients develop TKIs resistance, mediated by either *BCR::ABL1*-dependent or -independent mechanisms. Among the BCR::ABL1 dependent mechanism, the emergence of point mutations within the *BCR::ABL1* kinase domain (KD) is a well-characterized cause of resistance [20,21]. These mutations can impair TKI binding, and their identification allows for a tailored therapeutic approach based on the sensitivity profile to different TKIs. One clinically significant mutation, the T315I, often referred to as the “gatekeeper” mutation, confers resistance to the majority of currently available TKIs. The T315I mutation is rarely detected in patients with CP_CML, whereas it is more commonly found in patients with resistant disease of in BP. In this review, we provide an update overview of the current therapeutic strategies targeting *BCR::ABL1* T315I mutation. We will also focus on the role of the third-generation TKIs and discuss emerging investigational molecules with potential to overcome this resistant mutation.

## 2. Chronic Myeloid Leukemia Treatment

In recent decades, the therapeutic management of CML patients has been primarily based on TKI therapy. The therapeutic goals in the management of CML aim at improving overall survival and achieving a sustained deep molecular response (DMR). A stable and sustained DMR may allow for treatment-free remission (TFR), minimizing both short- and long-term toxicities [22,23,24]. Different TKIs have been tested, and ongoing research continues to focus on the development of novel agents. TKIs are administered orally on a daily schedule and induce their maximum therapeutic effect gradually over time. Tumor burden typically decreases progressively and should be monitored regularly through appropriate molecular assessments according to the International Scale (IS) [20,22,24]. According to the European LeukemiaNet (ELN) recommendations, a *BCR::ABL1* transcript level ≤ 0.1% is defined as major molecular response (MMR) or MR3, a *BCR::ABL1*^IS^ transcript level ≤ 0.01% with >10,000 ABL1 transcripts is considered MR^4^, whilst a *BCR::ABL1*^IS^ transcript level ≤ 0.0032% with >32,000 ABL1 transcripts is an MR^4.5^ [20,25,26].

**Figure 1:**
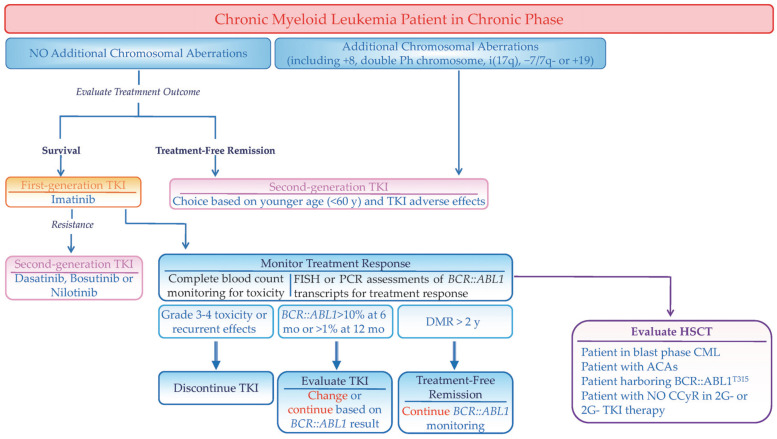
*Schematic representation of a therapeutic algorithm for patients with CML in chronic phase.* CCyR = Complete Cytogenetic Response; DMR = Deep Molecular Response; FISH = Fluorescence In Situ Hybridization; HSCT = Hematopoietic Stem Cell Transplant; TKI = Tyrosine Kinase Inhibitor (Imatinib, Nilotinib, Bosutinib, Ponatinib and Asciminib).

To date, five *BCR::ABL1* TKIs have been approved by the US Food and Drug Administration (FDA). Among these, Imatinib (IM), Dasatinib (DAS), Nilotinib (NIL), Bosutinib (BOS) and Asciminib (ASC) are FDA-approved for the frontline therapy, while all six agents, including Ponatinib (PON), are utilized in later-line treatment setting [20]. Figure 1 describes a treatment algorithm for CP-CML patients.

Moreover, according to the ELN recommendations for treating newly diagnosed CML patients, the 1G-TKI IM and 2G-TKI DAS, NIL and BOS are considered the best options for first line treatment [20]. DAS is a Src family of kinase inhibitor and is 350 times more potent than IM in vitro, NIL is a structural analog of IM with affinity for the ATP-binding site and is about 30–50 times more than IM, while BOS is a potent dual Src/ABL kinase inhibitor [20]. Recently, ASC, which is a potent allosteric inhibitor of ABL and BCR::ABL1 and a non ATP-competitive TKI, since binds to the myristate-binding pocket (a distinct and separate region of the kinase domain), received FDA approval for both first line CML treatment and for patients with the T315I mutation (at the increased dose of 200 mg BID dose) in October 2024 [27,28,29]. Different studies demonstrated that the long-term administration of IM for CML is safe and effective, with an estimated 10-year overall survival rate of 83.3%, resulting in high rates of complete cytogenetic response (CCyR = 91.8%), with 93.1% achieving MMR [30]. The DASISION trial enrolled 519 newly diagnosed individuals with CML-CP and compared IM with DAS [31,32,33]. Results from the 5-year follow-up demonstrated that DAS induced more rapid cumulative and deeper molecular responses (MMR = 76%, and MR^4.5^ = 42%) as compared with IM (MMR = 64%, and MR^4.5^ = 33%) (*p* = 0.0022 and *p* = 0.0251, respectively). In this trial, the T315I mutation was identified in eight patients treated with DAS and was not detected in IM-treated. Subsequent studies have confirmed the superiority of DAS as compared to IM, even when using a lower DAS dose, which was also associated with a lower incidence of adverse events (AEs) [34,35,36]. The ENESTnd trial compared two doses of NIL (300 mg or 400 mg twice daily) with IM 400 mg once daily. With a median follow-up of 10 years, cumulative rates of both MMR and MR4.5 were higher with NIL (300 mg twice daily, 77.7% and 61.0%, respectively, and 400 mg twice daily, 79.7% and 61.2%) than with IM (400 mg once daily, 62.5% and 39.2%). In addition, the estimated 10-year progression-free survival (PFS) rates were 86.2% with NIL 300 mg, 89.9% with NIL 400 mg and 87.2% with IM [37]. In the ENESTnd trial, nine patients developed the T315I (4 in NIL 300 mg arm, 2 in NIL 400 mg and 3 in IM treated patients) in the first five years of follow-up. The BFORE trial enrolled 536 patients with newly diagnosed CML-CP and compared BOS (400 mg once daily) with IM at conventional dose. Final trial results at 5 years showed cumulative rates of MMR higher with BOS than IM (73.9% vs. 64.6%) as well as of MR4 (58.2% vs. 48.1%) and MR4.5 (47.4% vs. 36.6%), while the 5-year overall survival rates were similar (94.5% vs. 94.6%) [38,39]. The T315I mutation was detected in six patients (five treated with BOS and one treated with IM).

In cases of resistance to frontline therapy with IM, a treatment change is necessary and the presence of BCR-ABL1 mutations must be investigated. All 2G-TKIs could be effective and the criteria for the choice are patient-related. This situation can also depend on age, comorbidities, previous toxicities, and, obviously, by presence of any mutations. Once a patient develops resistance to a 2G-TKI, whether in the second or later lines of therapy or even during frontline treatment, switching to another 2G-TKI is generally not recommended [40]. Consequently, to overcome resistance mechanisms associated with earlier-generation TKIs, the 3G inhibitors were developed. To address this point, PON [41] a pan BCR::ABL1 TKI was developed with the property of overcome the T315I mutation. PON received approval based on the results of the phase II PACE trial, which recruited 449 patients with heavily pre-treated CML or Ph + ALL patients, among which 270 were CP-CML affected patients. Overall, with 5 years follow up, 60% of CP-CML affected patients achieved major cytogenetic response and approximately 40% reached at least an MMR [42]. In this trial, sixty-four of the CP-CML patients displayed the T315I mutation at study entry and 70% of them achieved a CCyR whilst 58% gained MMR. Recently, the ASCEMBL study compared ASC with BOS in patients with CP-CML post-failure after ≥2 TKIs treatments [43]. This a phase III trial recruited 233 patients, 157 received ASC therapy (40 mg twice daily) while 76 had BOS (500 mg once daily). The follow up analysis after 4 years showed an MMR rate of 33.8% with ASC treated as compared to 10.5% with BOS, with a non-improved 2-year PFS rate (94% vs. 91%) and 2-year OS rate (97% vs. 99%). None of the patients exhibited the T315I mutations at baseline, while two patients developed it during treatment (one with ASC and one with BOS) and discontinued treatment (Table 1).

Hence, although an increasing number of patients achieve and sustain a *BCR::ABL1* clearance on 2G- or 3G-TKIs, adherence to therapy remains critical to maintain a long-term treatment efficacy. Moreover, all TKIs are associated with hematologic toxicities, along with agent-specific adverse effects, such as pleural effusion, or arterio-occlusive events, peripheral artery disease and gastrointestinal disturbance. Therefore, allogeneic hematopoietic stem cell transplant (HSCT) might represent a therapy option, with cure rates ranging 20–60% and depending on the disease phase at the time of transplant. According to ELN recommendations, stem cell transplant should be typically considered in eligible patients in CP who are resistant or intolerant to multiple TKIs, to a 2G-TKI treatment, to PON and/or ASC therapies [20], as well as those in AP or BP CML [15,16].

## 3. Mechanisms of Drug Resistance in Chronic Myeloid Leukemia

The development of 2G- and 3G-TKIs had the target to overcome resistance mechanisms to IM. Particularly, resistance may be associated with genomic amplification, overexpression of *BCR::ABL1* transcripts, additional chromosomal aberrations and mainly point mutations in the *ABL1* tyrosine kinase domain [44]. Thus, TKIs like NIL, DAS, BOS, PON and ASC can defeat some resistant mutations, although CML stem cells remain a challenge due to their reliance on multiple survival pathways [45].

*BCR::ABL1* monitoring and mutation analysis are crucial for managing resistance, with an individualized treatment based on mutation analysis and patient comorbidities. In the past, conventional Sanger sequencing was the recommended technique for diagnosing *BCR::ABL1* KD mutations. However, this method suffers from several limitations. Its sensitivity is restricted (it cannot reliably detect mutations present in less than 15–20% of transcripts) and, in many instances, it fails to provide clear distinction between polyclonal and compound mutations. To address these shortcomings, several Next-generation sequencing (NGS)-based approaches have been recently developed and described. These advanced techniques allow for the sensitive detection and precise quantitative tracking of *BCR-ABL1* KD mutations. They can detect and measure sequence variations in *BCR-ABL1* transcripts with a detection limit as low as 1–5% abundance, offering the capability to define the clonal landscape in most patients with multiple mutations [46]. The use of allele-specific oligonocleotide-reverse transcriptase polymerase chain reaction (ASO-RT-PCR), a highly sensitive methods, can be useful when the mutation is knows and must be confirmed. Particularly, point mutations in the *ABL1* kinase KD, which impair TKI binding through different mechanisms, are observed in approximately one-third of individuals exhibiting resistance to first-line treatment and in up to 50% of those resistant to second- or later-line of therapy [46]. Notably, they are more frequently associated with acquired resistance, which emerges after an initial treatment response [46,47,48]. Point mutations may alter the conformation of *BCR::ABL1*, reducing its affinity for the inhibitor, or may directly interfere with the drug-binding site, preventing effective inhibition [49,50]. They can arise in different structural regions of the KD, each playing a distinct role in resistance. Some mutations may occur at the TKI binding site, directly affecting the interaction between the drug and the kinase. Others are found in the phosphate-binding loop (P-loop), a critical region for ATP binding, where mutations are often associated with high levels of drug resistance. The activation loop (A-loop), which regulates kinase activity, is another key site where point mutations can influence drug response. Similarly, changes in the catalytic loop (C-loop), essential for enzymatic function, can contribute to resistance by altering kinase activity [49,50]. Historically, the presence of T315I and P-loop mutations, like G250E, Q252H, Y253H/F, E255K/V, correlated with significantly reduced PFS and OS [21,51]. Therefore, each TKIs exhibits a distinct sensitivity profile to specific *BCR::ABL1* mutation and the identification of the type play a critical role in driving the choice of the most appropriate TKI following the failure of first or subsequent lines of treatment [51]. Additionally, the presence of compound *BCR::ABL1* mutations is significantly high in late CP-CML patients and are associated with disease progression [52]. Also, DAS-treated patients exhibited a narrower spectrum of *BCR::ABL1* mutations compared to patients treated with IM, with lower phosphate-binding loop mutations and a higher occurrence of the T315I mutation [50].

## 4. The T315I Mutation

**Figure 2:**
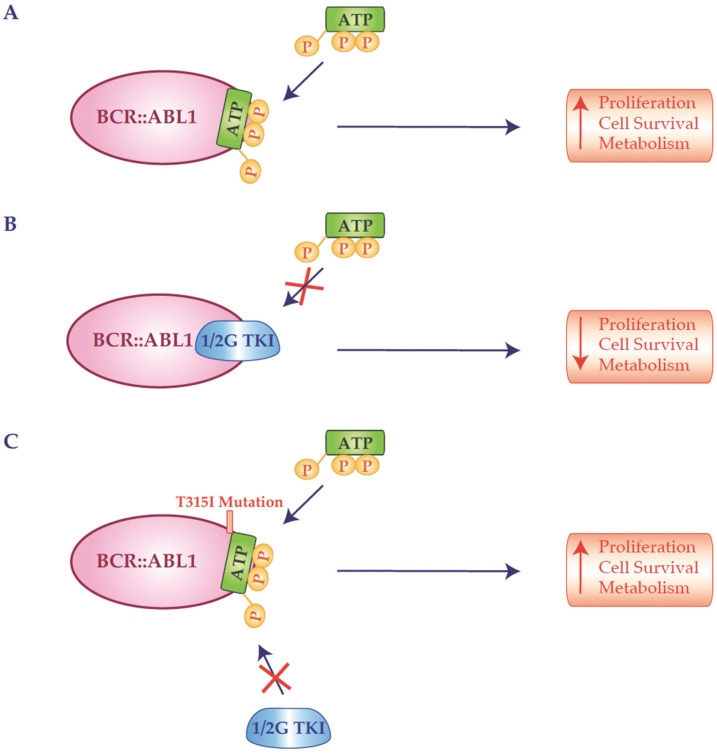
Schematic representation of structural mechanism of ATP or tyrosine kinase inhibitor binding to the BCR::ABL1 kinase domain (KD) and the impact of T315I mutation. (**A**) The BCR::ABL1 tyrosine kinase binds ATP within its catalytic cleft, located between the N-terminal lobe and C-terminal lobe of KD. ATP interacts with key residues in the phosphate-binding loop (P-loop) and the activation loop (A-loop), enabling phosphate transfer to substate proteins and activation of downstream signaling pathways that promote proliferation, cell survival and metabolism. (**B**) First (1G) or second generation (2G) TKIs occupy the ATP-binding pocket in the inactive conformation of the KD, establishing hydrogen bonds with hinge region. (**C**) The T315I mutation, which replaces threonine with a bulkier isoleucine, disrupts the critical hydrogen bond with inhibitors and introduces steric hindrance within the ATP-binding site. This conformational change prevents binding of both 1 and 2 G TKIs.

Among the different known mutations, the T315I mutation represents a truly challenging issue. The T315I mutation involves the gatekeeper residue threonine 315 (Thr315), located at the nucleotide-binding site of *ABL1*. This residue plays a key role in forming hydrogen bonds with IM [53]. A computational analysis revealed that the substitution of threonine with isoleucine at codon 315 of exon 6 in the *ABL1* gene induces a structural alteration in *BCR::ABL1*, particularly at the ATP-binding site. The mutation disrupts a critical hydrogen bond necessary for high-affinity binding of TKIs and introduces steric hindrance, thereby weakening the interaction with several TKIs and leading to drug resistance (Figure 2). In particular, the T315I alteration confers resistance to 1G-TKI (IM) and 2G-TKIs (DAS, NIL, BOS), while 3G-TKI inhibitors such as PON and the recently approved ASC have been specifically designed to retain efficacy against this mutation [28,53,54]. Liu J. and colleagues suggested that the inability to form a key hydrogen bond with Thr315 is not the sole cause of resistance. The mutation appears to induce also structural changes in the three-dimensional conformation of the enzyme’s catalytic center, accommodating the altered amino acid. Additionally, the T315I mutation modifies the enzyme cavity and affects interactions between Thr315 and residues Glu286 and Met290 [53].

Indeed, in a study evaluating the T315I mutation frequency, the alteration was identified in 7% of IM-resistant CML patients and was associated with advanced disease phases by using allele-specific (ASO) -RT-PCR assay. ASO-RT-PCR is a cost-effective method for screening the T315I mutation; however it may assist in therapeutic decisions [55]. Yusoff and collaborators investigated the prevalence of T315I in Malaysian patients and detected it in 5.26% of the subject resistant to IM, being also present across the three different CML phases [56].

Overall, according with a panel of experts appointed by the ELN in 2011, the presence of mutations can be investigated with NGS technique and recommended it for detecting *BCR::ABL1* KD mutations in CML patients with a “failure” or “warning” response to TKI therapy [21]. This most important technology can identify low-level mutations missed by Sanger sequencing. The consensus expert panel provided also recommendations for the use of NGS in clinical decision-making for CML, emphasizing its utility in guiding therapeutic changes [46]. Additionally, ultra-deep sequencing detects the T315I mutation in CML earlier and more sensitively than Sanger sequencing. Earlier detection of T315I mutations by ultra-deep sequencing may allow for timely treatment changes before clonal expansion [57]. Furthermore, new techniques such as the Fourier transform infrared (FTIR) micro-spectroscopy can rapidly and directly identify a spectral signature in single cells expressing T315I-mutated BCR-ABL. This method could serve as a novel early detection tool for mutant clones in leukemic cells [58] (Table 2).

## 5. Strategies and Treatment Options for T315I Mutation

Treatment strategies to overcome the T315I mutation may be different depending on the CML stage. Patients with CP-CML at the time of T315I mutation detection usually have better survival outcomes as compared to those in AP or BP [59]. To date, PON and ASC are the only TKIs indicated for the treatment of the T315I mutation in CP-CML by FDA [59,60], although the value of switching to ASC in cases of PON resistance and vice versa is less clear. Therefore, eligible CML patients should be referred for HSCT at the time of resistance to either. Hence, the phase of the CML disease at T315I detection seem to a more critical factor for survival than the presence of the mutation itself [20]. One of the most effective treatments is HSCT for CML patients with the T315I mutation, particularly when performed in CP. Patients who underwent HSCT in CP achieved long-term remissions and remained alive and in complete molecular remission [20]. In addition, Allogenic-HSCT (Allo-HSCT) can be considered the only curative treatment for CML patients harboring the T315I mutation [61]. CML patients in CP and AP at the time of HSCT show more promising survival rates, with 2-year leukemia-free survival rates of 80.0% and 72.9%, respectively [62]. Besides these treatments, which remain the most effective approach to prolong survival in these cohort of patients, salvage therapy with PON or CAR-T cells has proven helpful in *BCR::ABL1* positive acute lymphoblastic leukemia, serving as a bridge to allo-HSCT and improving overall survival [63]. Furthermore, PON showed high response rates and strong survival outcomes in CP-CML patients resistant to 2G-TKIs, including those with the T315I mutation [64]. Indeed, PON was created to address the need for a TKI able to effectively target the T315I mutation, while also inhibiting native *BCR::ABL1* and other frequently occurring resistance-associated mutations. Unfortunately, in long-term use, cardiotoxic effects can arise [65]. To reduce the cardiovascular risk associated with PON treatment, a response-based dosing strategy has been proposed to improve treatment tolerance as compared to fixed-dose strategies. The OPTIC trial demonstrated that a strategy on response-based dose-reduction resulted in lower related adverse events and longer median time on therapy than observed in the PACE trial [66]. In fact, in the PACE trial about 24% of CP-CML patients carried the T315I mutation at baseline, with nearly 70% achieving major cytogenetic response within 12 months and maintaining survival outcomes comparable to resistant/intolerant patients without the mutation. In addition, PON treatment significantly improved overall survival in patients with CP-CML as compared to allo-HSCT. Despite this, there was no significant difference in overall survival between PON and allo-HSCT for patients with accelerated-phase CML [67]. Other strategies were also investigated. Schneeweiss-Gleixner and colleagues studied the effects of combining hydroxyurea (HU) with PON on CML clones expressing *BCR-ABL1^T315I^* through in vitro experiments [68]. These authors observed that HU inhibited cell cycle progression in leukemic cells, accompanied by a reduced expression of CDK4 and CDK6. Both HU and the CDK4/CDK6-blocker palbociclib demonstrated to suppress the growth of CML clones harboring *BCR::ABL1^T315I^* or complex compound mutations including T315. A combination therapy with DAS and IFN-α was reported to achieve a deep molecular response in a CML patient carrying the T315I and E255V mutations. This combination therapy demonstrated as a potential alternative for patients who cannot use ponatinib or undergo allogeneic transplantation [69]. Likewise, Zhang Y et al. reported a case of T315I-mutated myeloid sarcoma occurring after a complete cytogenetic response to DAS in a patient with CP-CML. The successful treatment with induction chemotherapy combined with ponatinib suggested that this therapeutic approach may effectively induce remission in cases of CML-BP and associated myeloid sarcoma [70].

More recent studies reported that ASC is a highly effective in showing inhibition against T315I mutation. ASC is an FDA-approved allosteric inhibitor effective for CP-CML patients resistant or intolerant to ≥2 prior TKIs or with the T315I mutation [71,72]. ASC demonstrated long-term safety and efficacy in CML-CP patients without T315I mutations, with 69.6% remaining on treatment after approximately four years [72]. ASC is effective against several *ABL1* kinase domain mutations, including T315I, but resistance due to specific point mutations has been observed [28,72,73]. ASC treatment drove also to a sustained complete molecular remission a CML patient with an atypical e19a2 *BCR::ABL1* transcript and T315I mutation, also achieving treatment-free remission for 18 months after discontinuing ASC [74].

## 6. Possible Future Scenarios for Treatment

Despite remarkable outcomes in CML, a subset of patients develops TKIs resistance mediated by either *BCR::ABL1*-dependent or -independent mechanisms. Notably, BCR::ABL1-independent resistance in PON-resistant CML cells has been linked to mammalian target of rapamycin (mTOR) pathway activation. In a study conducted by Mitchell et al., PON-resistant cell lines [75], exhibiting BCR::ABL1-independent resistance, were treated with NVP-BEZ235, a dual phosphoinositide 3-kinase (PI3K)-mTOR inhibitor. Treatment with catalytic mTOR inhibitors led to autophagy induction, and the authors demonstrated that pharmacological inhibition of autophagy sensitizes PON-resistant CML cells to death induced by mTOR inhibition both in vitro and in vivo system.

**Figure 3:**
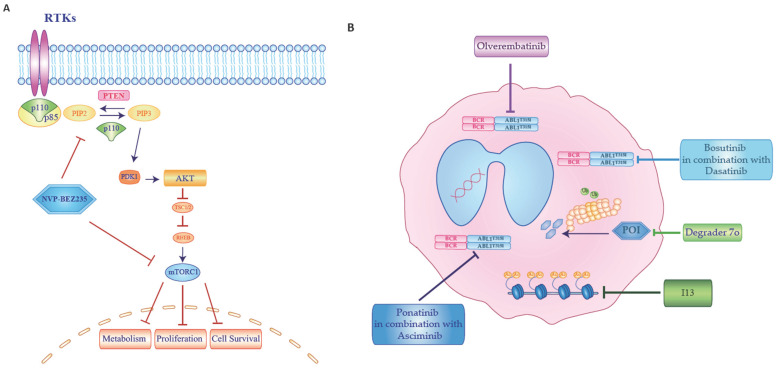
*Potential future therapeutic strategies for patients with Chronic Myeloid Leukemia harboring BCR::ABL1^T315I^ mutation*. (**A**) The dual phosphoinositide 3-kinase (PI3K)-mTOR inhibitor NVP-BEZ235 induced CML cell death. (**B**) Five different treatment strategies for CML patients with T315I alteration.

These finding suggest that the combination of mTOR and autophagy inhibition may enhance therapeutic efficacy in resistant CML cells. Catalytic mTOR inhibitors, particularly in combination with autophagy blockers, may represent a promising strategy for overcoming resistance in this patient population (Figure 3A) [75].

Among the BCR::ABL1 dependent mechanism, different strategies are currently under investigation to overcome the gatekeeper T315 alteration, which may represent potential future treatment options for patients with resistant CML. An open-label, multicenter phase 1/2 trial investigated the new 3G-TKI Olverembatinib [76], that is an ATP binding-site inhibitor. The study demonstrated the high efficacy of the drug in achieving cytogenetic and molecular responses in patients with TKI-resistant CML, particularly those with a single T315I mutation (Figure 3B). The drug was well tolerated, with manageable adverse events, making it a promising treatment option for TKI-resistant CML. Among these strategies, three separate studies have investigated the combination of two approved TKIs. In the first study [77], an Austrian group explored the combination of BOS and DAS in different cell lines (K562, K562R, KU812, KCL22, *BCR::ABL1* mutant Ba/F3) and in primary CML cells that were obtained from the peripheral blood from 23 CML patients (Figure 3B). The authors found a synergistic effect in inhibiting growth and inducing apoptosis in CML cells, including those carrying *BCR::ABL1^T315I^* and synergistic apoptosis-inducing effects on CD34+/CD38− CML stem cells. In second research, the authors analyzed the association of PON and ASC in both primary patient-derived CML cell lines and in *BCR::ABL1* mutated cell lines [78]. They found that ASC synergizes with PON because induced growth-arrest and apoptosis in both primary cell lines and murine Ba/F3 cells allowing *BCR-ABL1^T315I^* or T315I-including compound mutations (Figure 3B). Indeed, the combination of ASC, PON and HU synergized in producing anti-leukemic effects in multi-resistant CML cells. Another study investigated the combination of PON with ASC in a murine model of CML blast crisis harboring compound mutations [79]. The combination of the two therapies proved potent in suppressing tumor growth in the preclinical animal model. Further, different new molecules seem promising in preclinical studies. Among them, the I13, a potent histone deacetylase (HDAC) inhibitor, shows strong activity against T315I-mutated and wild-type BCR::ABL1-expressing CML cells, overcoming resistance issues (Figure 3B). The I13 inhibitor induces cell differentiation and suppresses proliferation through G0/G1-phase cell cycle arrest and can deplete *BCR::ABL1* expression, blocking its function and modulating the chronic myeloid leukemia signaling pathway [80]. Moreover, Kawakami and colleagues studied the antileukemic effects of a natural methylated polyphenol analog of resveratrol named Pterostilbene, which is predominantly found in berries and nuts [81]. The authors demonstrated that Pterostilbene effectively induces apoptosis in human and murine *BCR::ABL1* positive leukemic cells, including TKI-resistant T315I mutations. Also, the treatment reduced BCR::ABL1 protein levels and inhibited AKT and NF-κB activation. In vivo study with oral administration of pterostilbene significantly suppressed tumor growth in a mouse model of *BCR::ABL1* positive leukemia.

Other than the inhibition of BCR-ABL1 kinase activity, an alternative approach to treat CML-resistant cells is to degrade the BCR::ABL1 protein. In the recent years, a Chinese group investigated a new class of selective BCR::ABL^T315I^ proteolysis-targeting chimeric (PROTAC) degraders. Among them, the degrader 7o, carrying a six-member carbon chain linkage, displayed a potent degradation efficacy against *BCR::ABL^T315I^* in vitro and in vivo models (Figure 3B). The compound 7o significantly suppressed tumor growth in a Ba/F3 *BCR::ABL^T315I^* xenograft model, achieving a tumor growth inhibition value of 90.8% [82]. In this context, a study analyzed the interaction between protein-tyrosine phosphatase 1B (PTP1B), which is required for the stabilization of BCR::ABL1 protein, and BCR::ABL1 with SBF-1. This interaction led to the degradation of the BCR::ABL1 protein in CML cells, including those harboring T315I mutation. Therefore, targeting PTP1B-BCR::ABL1 interaction represent a potential therapeutic approach for TKI-resistant CML [83]. Finally, Quezada Meza et al. examined the role of protein kinase CK2 as a potential novel therapeutic strategy to overcome the T315I mutation-mediated TKI-resistance. The authors demonstrated that the inhibition of CK2 by the CK2-inhibitor CX-4945 (currently in clinical trials for the treatment of different tumors) reduces aberrant signaling, induces apoptosis, and decreases proliferation in T315I-mutated CML cells, thereby sensitizing them to TKI drugs [84,85].

## 7. Conclusions

CML has undergone a paradigm shift in CML treatment with the advent of TKIs, significantly improving patient outcomes. However, resistance mechanisms, particularly those involving *BCR::ABL1* mutations, continue to be a major challenge. Among these, the T315I mutation remains a critical point due to its resistance to first- and second-generation TKIs and necessitating alternative therapeutic strategies. PON has emerged as a key third-generation TKI effective against T315I, yet its long-term use is limited by cardiotoxicity. ASC, a novel allosteric inhibitor, offers another promising approach, though resistance mechanisms have been reported yet. Allo-HSCT remains a curative option, particularly for patients in CP resistant to 2G- and 3G-TKI, or intolerant to all available TKIs, and for those who present in or progress to BP. Additionally, novel therapeutic combinations, such as mTOR inhibitors and new TKIs such as Olverembatinib are under investigation to address resistance. Indeed, future treatment strategies will likely involve a multi-targeted approach, such as add-on TKIs, or integrating TKIs with epigenetic modulators, metabolic pathway inhibitors, and immune-based therapies, to enhance responses and overcome resistance. Additionally, advances in next-generation sequencing and early detection methods may optimize treatment decisions, for a more personalized and effective management of CML patients harboring the T315I mutation.

## Figures and Tables

**Table 1:** Key clinical trials in CML, including total patient enrollment, presence of T315I at baseline, incidence of mutation development while on therapy, response rates, and relevant clinical context.

Study Name	Total Patients Enrolled	Patients with T315I Mutation at Baseline	Patients Who Developed T315I During Treatment	Clinical Context/Notes
**IRIS**	1106	Not captured		Pivotal trial with IM
**DASISION**	519	Emergent	8 patients developed T315I	
**ENESTnd**	846	Emergent	9 patients developed T315I (4 in NIL 300, 2 NIL 400 and 3 IM treated patients)	Not effective for T315I mutation.
**BFORE**	536	Emergent	6 patients developed T315I (5 in BOS treated and 1 with IM)	BOS not effective for T315I mutation
**PACE**	449	64	T315I present in 64 patients at baseline	Ponatinib effective in resistant CML including T315I mutation
**ASCEMBL**	233	2	Not present at baseline in CP-CML	

**Legend: T315I** = Threonine-to-Isoleucine mutation at position 315 of BCR-ABL gene, conferring resistance to many TKIs.

**Table 2:** Molecular characteristics of the T315I mutation in CML with current detection methods.

Detection Method	T315I Mutation Characteristics	Mechanism/Principle	Reported T315I Prevalence/Context	Advantages/Limitations
**ASO-RT-PCR**	Gatekeeper mutation at codon 315 (Thr → Ile), disrupts hydrogen bond with TKIs, steric hindrance causing resistance to 1G/2G TKIs.	Allele-specific oligonucleotide reverse transcriptase PCR targeting codon 315 mutation	7% in imatinib-resistant CML; linked to advanced phases	Cost-effective, accessible; may miss low-level mutations
**ASO-RT-PCR (Malaysia study)**	Same mutation features: observed across all CML phases, linked to resistance.	Same principle applied to Malaysian patient cohort	5.26% in imatinib-resistant CML; present across all CML phases	Simple, feasible in low-resource settings
**Next-Generation Sequencing (NGS)**	Detects structural alteration in ATP-binding site; mutation impacts interactions with Glu286, Met290.	Massively parallel sequencing of BCR::ABL1 kinase domain	Identifies mutations in “failure” or “warning” TKI response categories	High sensitivity; detects low-level mutations missed by Sanger sequencing
**Ultra-Deep Sequencing**	Identifies low-level T315I before clonal expansion; mutation modifies enzyme catalytic center conformation.	High-depth NGS enabling detection of rare mutant alleles	Detects T315I earlier than Sanger sequencing	Allows treatment change before clonal expansion; higher cost
**FTIR Microspectroscopy**	Detects unique spectral signature of T315I-mutated BCR-ABL1 in single cells.	Fourier-transform infrared spectral analysis at single-cell level	Identifies specific spectral signature in T315I-mutated leukemic cells	Rapid, direct, non-destructive; novel technology requiring validation

## Data Availability

No new data were created or analyzed in this study. Data sharing is not applicable to this article.

## Abbreviations

1G	First Generation
2G	Second Generation
3G	Third Generation
A-loop	activation loop
C-loop	catalytic loop
P-loop	phosphate-binding loop
ABL1	Abelson1 gene
AKT	protein-chinasi B
ALL	Acute Lymphoblastic Leukemia
Allo-HSCT	Allogenic-Hematopoietic Stem Cell Transplant
AP	Accelerated Phase
ASC	Asciminib
ASCEMBL	sciminib vs. Bosutinib in Patients with Chronic Myeloid Leukemia in Chronic Phase
ASO-RT-PCR	allele-specific oligonocleotide-reverse transcriptase polymerase chain reaction
BCR	Breakpoint Cluster Region gene
BFORE	Bosutinib versus imatinib for newly diagnosed chronic phase chronic myeloid leukemia
BOS	Bosutinib
BP	Blastic Phase
CCyR	Complete Cytogenetic Response
CK2	protein kinase casein kinase 2
CML	Chronic Myeloid Leukemia
CP	Chronic Phase
DAS	Dasatinib
DASISION	Dasatinib Versus Imatinib Study in Treatment-Naïve Chronic Myeloid Leukemia Patients
DMR	Deep Molecular Response
ELN	European LeukemiaNet
ENESTnd	Evaluating Nilotinib Efficacy and Safety in Clinical Trials–Newly Diagnosed Patients
FDA	Food and Drug Administration
FISH	Fluorescence In Situ Hybridization
FTIR	Fourier Transform Infrared spectroscopy
HDAC	histone deacetylase
HSCT	Hematopoietic Stem Cell Transplant
HU	hydroxyurea
IM	Imatinib
KD	Kinase Domain
MMR	Major Molecular Response
MR^4.5^	molecular response with 4.5-log reduction
NIL	Nilotinib
PACE	Ponatinib Ph-positive acute lymphoblastic leukemia and CML Evaluation
PFS	Progression-free Survival
Ph	Philadelphia
PON	Ponatinib
PROTAC	proteolysis-targeting chimeric
PTP1B	protein-tyrosine phosphatase 1B
TFR	Treatment-Free Remission
TKI	Tyrosine Kinase Inhibitor
T315I	Threonine-to-Isoleucine mutation at position 315 of BCR-ABL1 gene
